# Design and optimization of thermal parameters of building envelope for senior living community in Chinese hot summer and cold winter area

**DOI:** 10.1038/s41598-025-94443-x

**Published:** 2025-03-25

**Authors:** Xilu Tang, Bing Han, Ziwen Mao, Wei Xu

**Affiliations:** 1 School of Design Art and Media, Nanjing University of Science and Technology, Nanjing, 210096 China; 2 College of Horticulture, Jinling Institute of Technology, Nanjing, 210038 China; 3 Unit 61175, People’s Liberation Army, Nanjing, 210042 China

**Keywords:** Senior living community Building, Simulation, Enclosure structure, Economic analysis, Civil engineering, Engineering

## Abstract

As living standards and medical services improve, the elderly population continues to grow, drawing attention to senior living communities. This study analyzes the load characteristics of buildings in five Chinese towns with hot summers and cold winters by simulating their annual cooling and heating loads, exploring the effects of wall insulation thickness and window glazing layers on thermal loads across regions. Through a life cycle cost analysis, this study identifies the most cost-effective wall insulation and window configurations for each location, offering valuable guidance for renovating senior living communities in such climates. The results indicate that annual cooling loads vary from 113.0 W/m² to 145.7 W/m², while heating loads range between 14.7 W/m² and 41.8 W/m². Specifically, Chongqing benefits most from single-glazed windows with a 0.06 m insulation layer, while Changsha, Hefei, Nanjing, and Shanghai opt for double-glazed windows with insulation layers of 0.08 m, 0.10 m, 0.10 m, and 0.06 m respectively for optimal cost-effectiveness. Notably, Shanghai has the lowest life cycle cost, which is about 30% less than that of the other cities. This is attributed to its lower summer cooling load. Overall, the minimum 25-year life cycle cost for upgrading air conditioning and enclosure structures falls between 148kRMB and 165kRMB.

## Introduction

Nowadays, the proportion of the elderly population is increasing rapidly, with a 2017 report indicating that the numbers of those aged 60 years or over today are more than twice the numbers in 1980^1,2^. Although most older people are reluctant to leave their own homes with their children^3^, their need for care, nutrition, and health is high and their children often have stressful jobs make their wishes are difficult to carry out^4^. Therefore, the senior living community may be one of the places for older people will live in the future. Indoor thermal environment has a significantly influence on the health and comfort of the resident^5^, especially when the senior people live at least 80% of their time in an indoor environment^6,7^. Therefore, it is very important that reasonably design a series of envelope paramenters to create a good indoor environment for senior people.

In the past, much attention has been paid to the environment needed by the senior people. Giamalaki and Kolokotsa studied the thermal sensation, preference and adaptive of the older in Crete, Greece^8^. They find that the senior people consider more important to heat their homes in winter than to cool them in summer. Tartarini et al. hold the opinion that the residents in nursing houses were more tolerant of temperature variations than staff or visitors^9^. Both the estimated neutral and preferred temperatures were higher for residents than for non-residents. Tejedor et al. proposed a method to determine the indoor thermal comfort of senior people by IRT during winter, estimating the thermal exchange from three body-segments (head, back-pelvis, thorax-limbs) to the surroundings^10^. Forcada et al. studied the thermal sensation of senior people during summer season in nursing homes from the Mediterranean climate, finding that the comfort temperature for senior people was 24.4 °C while for non-senior people was 23.5 °C. China is the country with the largest population in the world, and the thermal comfort of the senior people has also been studied by many scholars. In their study, Wu^11^, Wang^12^, and Yu^13^ observed that senior people in Chongqing, Beijing, and Hefei prefer natural ventilation over younger populations during summer months. Besides, research conducted in the Xi’an^14^ has revealed that the thermal neutral temperatures for elderly people are 19.4 °C, 22.6 °C, and 24.1 °C during winter, transition seasons, and summer, respectively. Additionally, studies in Shanghai^15^ have shown that the summer thermal neutral temperature for senior people ranges from 25.4 °C to 26.0 °C under different clothing conditions, while in winter, it varies between 16.6 °C and 21.6 °C. Their research similar found that the thermal comfort requirements of the elder were quirt different from the younger. The senior people often have higher acceptability and lower psychological expectations on the thermal environment. Furthermore, across various regions in China, senior people exhibit similar thermal neutral temperatures, demonstrating distinct characteristics compared to those of younger people. Therefore, the needs of the senior people for thermal environment must be concerned when designing a building envelope for senior living communities.

Besides, energy consumption is also an important factor in the design of a building envelope. There are various aspects of the world energy problem, requiring every countries to save energy and reduce emissions. In September 2020, it was announced that China will achieve a peak in carbon emissions (CEs) based on energy consumption by 2030 and carbon neutral (CN) by 2060^16^. In hot summer and cold winter area of China, air conditioning consumes a lot of energy in summer and winter^17^. Improving the insulation performance of the building envelope can contribute to reducing air conditioning energy consumption^18^. It is important to recognize that the optimal balance between thermal transmittance and thermal mass is highly dependent on the local climate. The vast territory of China encompasses a wide range of climates, necessitating region-specific solutions^19^. Due to variations in latitude and longitude, different regions within the same climatic zone may also yield different conclusions^20^.Therefore, how to reasonably design the thermal parameters of building envelope for senior living community is an important engineering problem.

In order to reduce the energy consumption of the building while providing a good living environment for the senior people, the annual cooling and heating loads of a typical building in senior living community are simulated. The annual energy consumption of the typical building is compared at five different cities in Chinese hot summer and cold winter area. With the increase of thermal insulation performance of building envelope, the cooling and heat load decreases, but the cost of the initial investment increases. Therefore, the optimal building envelope scheme is carried out through economic analysis.

## Method

### Calculation method of building heating and cooling loads

Numerous software tools, such as EnergyPlus^21^, TRNSYS^22^, and DEST^23^, are available for calculating the heating and cooling loads of buildings. However, due to unknown factors, some discrepancies exist among the results generated by different software, making it challenging to determine which one is more accurate. Utilizing the fundamental steady-state heat conduction equation to calculate loads appears to approximate the ideal scenario, representing the unlimited capacity for cooling or heating to maintain a stable indoor temperature. This approach merits calculation and discussion under any circumstances. Consequently, a steady-state heat conduction equation for the building is established using Excel, where each time point is assumed to be in steady-state conduction. Each row in the table calculated the load for a specific time point, totaling 8760 rows. The monthly and annual loads are then obtained by summing the respective values. The hourly cooling and heating loads are calculated using the lumped parameter method, and relevant papers^24–28^ are referenced for reference. The existing research shows that the calculation results of this method are similar to those of software (DOE-2, DeSH-h)^29^, and it is also widely used in the professional field.

The cold and heat load of the building is solved according to the energy balance equation of the building, shown in Eq. 1. The sum of the cold and heat load, the heat transfer by the envelope, the heat transfer by radiation through the glass, the heat dissipation by personnel, the heat dissipation by ventilation and infiltration, the heat dissipation by equipment, and the heat dissipation by lighting is equal to 0^24^. If the *Q*_0_ obtained by the formula is positive number, the *Q*_0_ represents the heat load of the building. If the *Q*_0_ obtained by the formula is negative number, then the |*Q*_0_| represents the cool load of the building.

1 $$0\,=\,{Q_0}\,+\,{Q_{{\text{body}}}}+{Q_{{\text{glass}}}}+{Q_{\text{P}}}+{Q_{{\text{air}}}}+{Q_{\text{E}}}+{Q_{\text{S}}}$$

In which, the *Q*_0_ represent the heat load or cool load of the building, W; the *Q*_body_ represent the heat transfer by the envelope, W; the *Q*_glass_ represent the heat transfer by radiation through the glass, W; the *Q*_P_ represent the body cooling load of people, W; the *Q*_air_ represent the heat dissipation by ventilation and infiltration, W; the *Q*_E_ represent the heat dissipation by equipment, W; the *Q*_S_ represent the heat dissipation by lighting, W.

The heating and cooling loads are calculated under steady-state conditions, with the thermal mass effects of building envelopes excluded from consideration. It is because the heat storage in building envelopes has minimal impact on annual cooling and heating load calculations. Although thermal inertia can produce both beneficial and adverse effects, these opposing influences largely offset each other. For example, during summer transitions from cooler to warmer periods, the relatively lower envelope temperature temporarily reduces initial cooling load demand until a new thermal equilibrium is established. Conversely, when ambient temperatures shift from higher to lower conditions, the residual heat stored in building components may paradoxically extend the required cooling operation time. More critically, the hourly thermal flux caused by material heat storage remains negligible when compared to the magnitude of typical cooling and heating loads.

### Typical cities

**Fig. 1:**
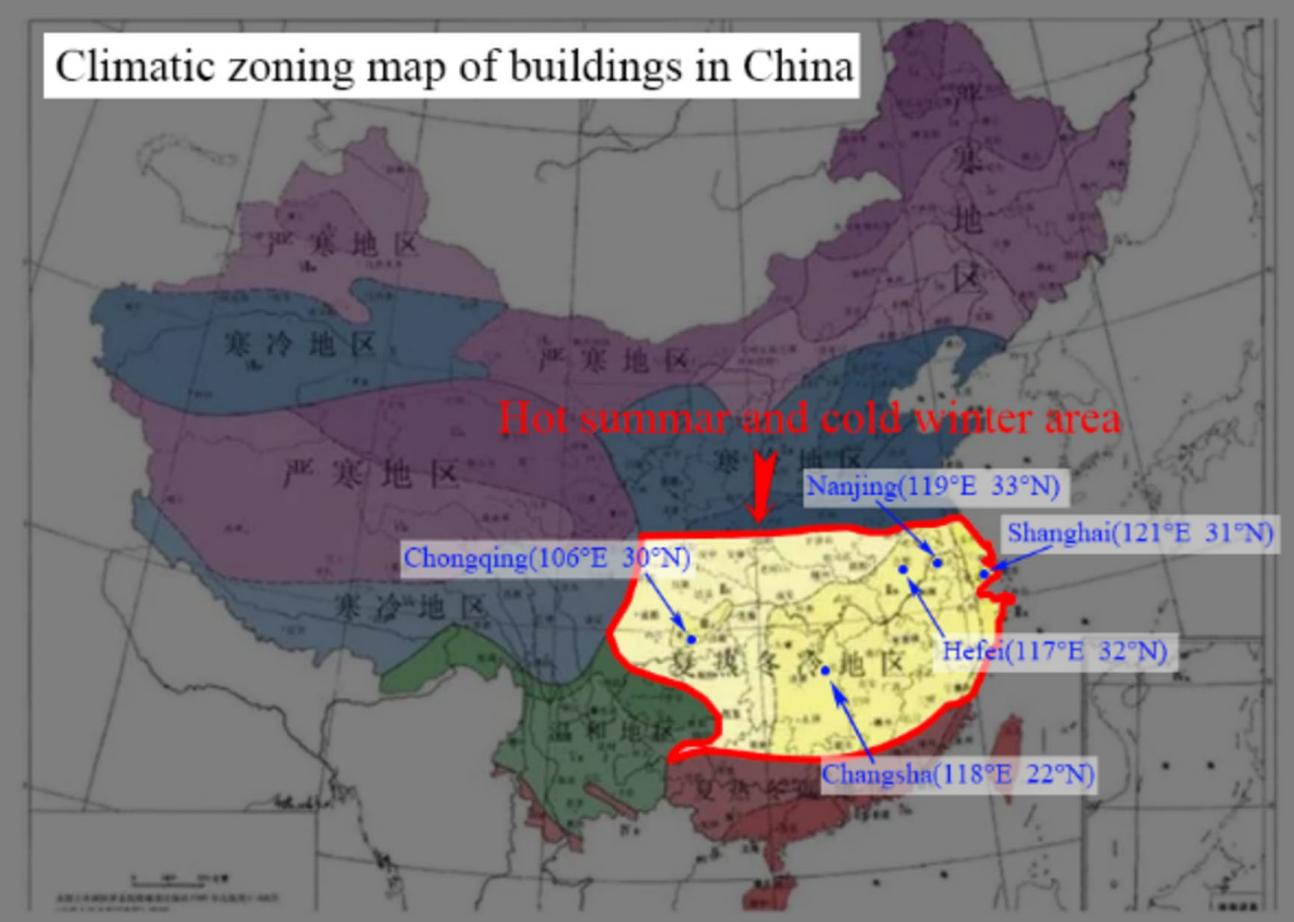
Five typicle city in hot summar and cold winter area.

Five typical cities (as shown in Fig. 1**)** are selected to comprehensively study the characteristics of hot summer and cold winter areas, where are Chongqing(106°E, 30°N), Changsha(118°E, 22°N), Hefei(117°E, 32°N), Nanjing(119°E, 33°N), Shanghai(121°E, 31°N). The five cities are are important large towns distributed in hot summer and cold winter area, which are important towns for future senior care community construction. The hourly meteorological parameter data files of these five cities are comes from the publicly website from Energy Plus, which are in CSWD format. The average horizontal solar radiation and the average temperature of the year, hottest month and coldest month are shown in Table 1. The largest solar radiation town is Shanghai, while the worst is Chongqing. The hottest town is Chongqing, while the worst is Nanjing. The Köppen-Geiger climate classification is also an important reference. According to the research by KOTTEK et al.^30^, all five towns mentioned in this paper belong to the Cfa region.

**Table 1:** The total horizontal solar radiation and the average temperature of the five cities.

City	Total horizontal solar radiation (MJ)			Average temperature (°C)		
	Year-round	Hottest month	Coldest month	Year-round	Hottest month	Coldest month
Chongqing	3053.9	480.9	93.2	18.5	28.1	8.1
Changsha	3905.7	568.6	168.4	17.1	28.5	5.5
Hefei	4159.1	458.2	197.6	16.2	28.0	3.0
Nanjing	4350.5	483.9	230.8	15.8	28.6	2.2
Shanghai	4576.8	487.9	226.1	16.7	27.5	4.5

**Fig. 2:**
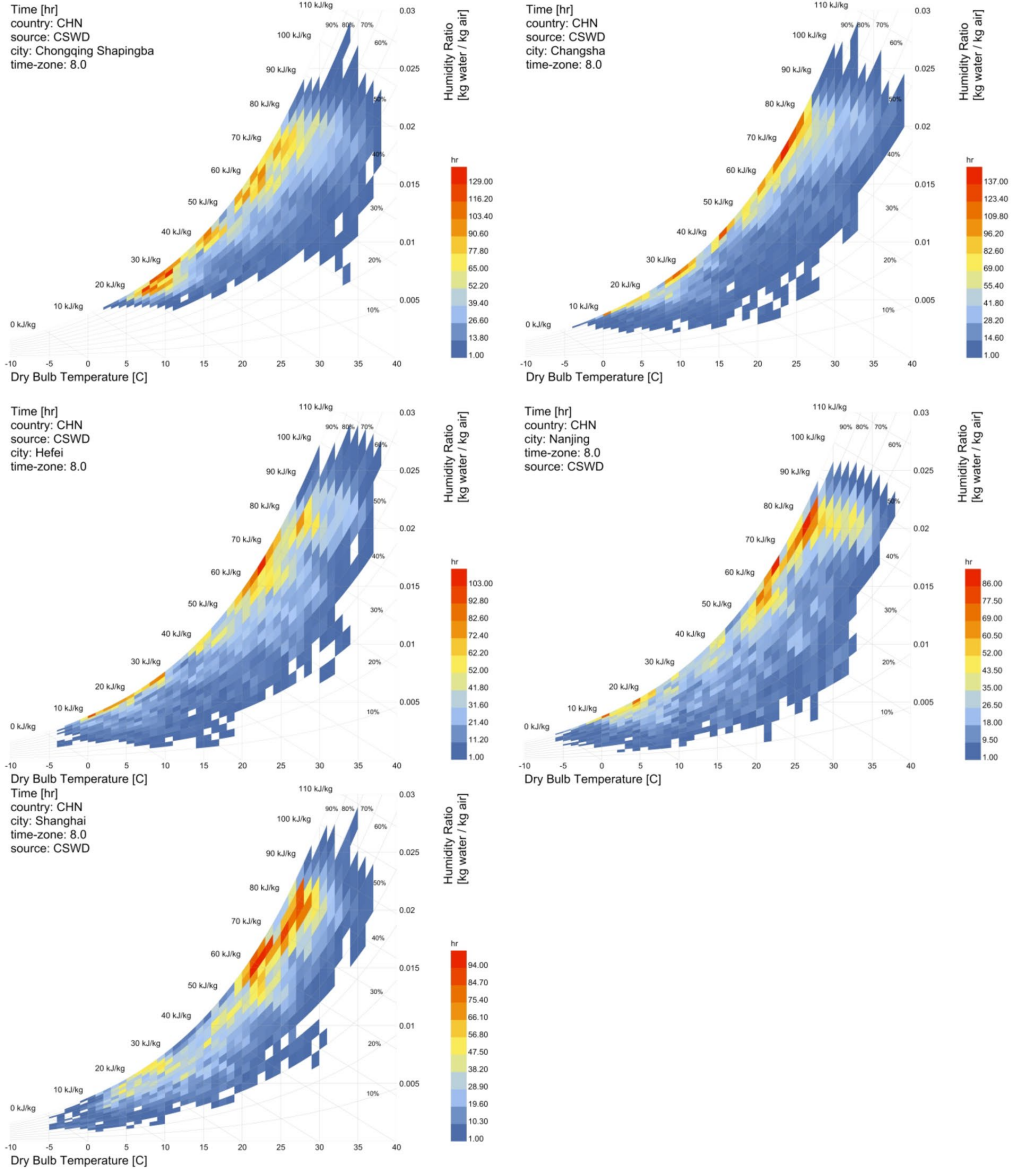
The psychrometric diagrams of the five cities.

Figure 2 presents the psychrometric charts of five Chinese cities (Chongqing, Hefei, Shanghai, Nanjing, and Changsha) derived from CSWD data (UTC + 8). Comparative analysis reveals distinct regional thermal-humidity patterns, with all cities demonstrating a positive correlation between dry-bulb temperature and humidity ratio at varying intensities. Chongqing shows significant humidity ratio fluctuations (ΔHR > 0.012 kg/kg) during high-temperature periods (30–38 °C) alongside sustained winter high humidity, characteristic of mountainous terrain-induced microclimates. Changsha and Hefei exhibit comparable patterns, with high-density hourly distributions concentrated in two temperature bands (20–25 °C and 0–15 °C) accompanied by persistently high relative humidity conditions. Changsha and Hefei exhibit comparable patterns, with high-density hourly distributions concentrated in two temperature bands (20–25 °C and 0–15 °C) accompanied by persistently high relative humidity conditions. Nanjing displays a predominant warm-humid zone (25–35 °C, RH 70–90%). Shanghai exhibits coastal climatic moderation, with 75% of data concentrated in a narrow marine-influenced band (20–30 °C, RH 75–90%, 1,200 + hours/year) and rare cold events. In summary, Chongqing exhibits pronounced humidity volatility, Shanghai demonstrates maritime climatic stability, and the inland cities display transitional bimodal patterns. These divergences necessitate region-tailored building energy strategies.

### Typical building in senior living community

**Fig. 3:**
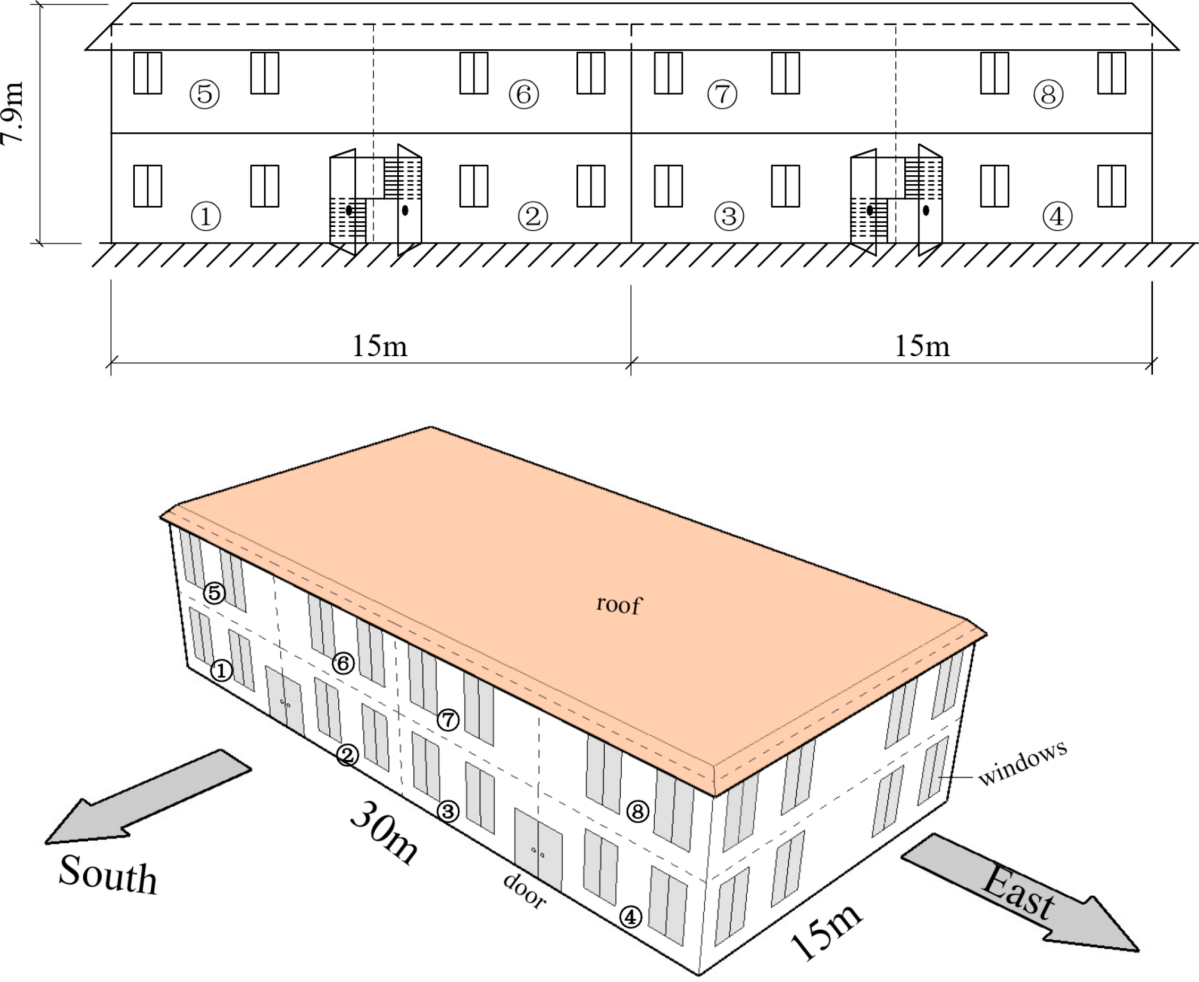
The typicle building.

The mobility of the senior people are often impaired, so the typical building is set at two stories, as shown in Fig. 3. There is a outdoor yard out side the building, but it has no heat and cold load. The building is situated in a suburban area of the city where the building density is relatively low. It is assumed that there are no tall structures surrounding the site that could obstruct sunlight throughout the day. Originally, this structure belonged to a small community and served as residential accommodation. However, in response to the ongoing demographic shift towards an aging population, modifications are planned to better suit the needs of elderly residents.

The building is divided into 8 rooms and two floor, the length, width and height of the building are 30.0 m, 15.0 m and 7.9 m respectively. The building is divided into 8 rooms and two floor, the length, width and height of the building are 30.0 m, 15.0 m and 7.9 m respectively. The loads of households at different building locations vary. Therefore, this study labels households in distinct positions and conducts further load analyses to demonstrate the differences in building loads caused by orientation. Accordingly, we have added room divisions and markings in Fig. 3, where circled numbers ①, ②, ③, …, ⑧ indicate the positions of the eight households. Among them, households 1 and 5 are located on the westernmost side, while households ④ and ⑧ are on the easternmost side. During load calculations, households ② and ③ share the same layout, as do households ⑥ and ⑦, resulting in identical loads.

The floor area is 900m^2^. The long axis of the building is indeed oriented east-to-west and providing predominantly South and North facades, as shown in the Fig. 3. This orientation is chosen based on the typical architectural form prevalent in Chinese hot summer and cold winter area. Each room accommodates two senior people, with an average living area of 56.3m^2^ per person. It is assumed that the ratio of men to women in the room is equally. The area of the east, west, south and noth walls are 88.5m^2^, 88.5m^2^, 177.0m^2^, 189.0m^2^, respectively, while the area of the east, west, south and noth windows are 30.0m^2^, 30.0m^2^, 60.0m^2^, 48.0m^2^ respectively. The overall window-to-wall ratio of the building is 0.236. To optimize natural lighting and shield against the cold northern winds in winter, the window area on the southern side is larger than that on the northern side. The structure of the wall is mainly including the inner and outer finish coating, concrete layer, waterproof layer and thermal insulation layer. The structure of the roof is similar with the wall, but the outer surface of the roof has an extra layer of tile. The heat insulation ability of walls and roof is mainly determined by the thermal insulation layer. Therefore, the thermal conductivity of the insulation layer is used to represent that of the wall or roof. Expanded polystyrene (EPS) are one of the most used thermal insulation layers, which has the advantages of the low thermal conductivity, small volume weight and small density. The thermal conductivity of EPS is 0.038 W·m^− 1^·°C^− 1^, and the EPS can work more than 25 years.

The somatic functions and living habits of the senior people are different from the other people. The differences in their living habits can be summarized in the following four points. Firstly, The senior people has a low thermal sensitivity so that their thermal neutral temperature is higher in summer but lower in winter. Secondly, the senior people do not need to work outside, so they spend most of their time at home during the hottest summer and coldest winter months. Thirdly, the metabolism of the senior people is low, resulting in their body heat load is small. Fourthly, the senior people like to cool down by opening windows for ventilation in summar.

The parameters in the load calculation process must conform to the definition of the senior people. Firstly, the indoor design temperatures in winter and summer should be adjusted according to the results of the existing literatures. The indoor design temperatures equals the neutral sensation temperature of the old people, which are 29.3 °C in summar and 13.4 °C in winter^15^. Secondly, when the building is needing heating or cooling, all senior people are indoors to ensure that their surroundings are comfortable. And the senior peoplr constantly emit heat to the buildings. Thirdly, an adult man has a combined sensible and latent heat load of approximately 108 W while seated^31^, and the heat load of woman is about 85% of that of man. The heat dissipation of human body is related to oxygen consumption. Compared with the age of 30, the oxygen consumption of both men and women at the age of 70 decreases by about 30%^32^. Therefore, the total heat load of senior man and woman are 75.6 W and 64.3 W, respectively. Fourthly, when the outdoor temperature is lower than the indoor temperature in summer, the windows are opened for ventilation. The natural ventilation can be accurately modeled by considering infiltration rates and air exchange dynamics^33^. Nevertheless, to accommodate the habits of Chinese senior people, the calculation model of the natural ventilation is simplified. The natural ventilation rate is determined through multiplying the wind speed by the window opening area, where the outdoor wind speed can be directly retrieved from the hourly meteorological data files, and the window opening area is deemed to be half of the total window area. However, when the outdoor wind speed exceeds 5 m/s, people may feel uncomfortable and take measures such as closing windows or using shields to reduce the airflow. Therefore, in this study, it is assumed that the maximum wind speed for natural ventilation is limited to 5 m/s.

The other initial parameters used for simulation are designed with reference to the current standards of China^31^, and these parameters are shown in Table 2, where ‘*v*’ denotes the outdoor wind speed. Any modifications made to the windows or walls mentioned subsequently are based on these initial parameters. Since the temperatures in all rooms within the house are assumed to be the same, the thermal conductivity of internal walls and internal floors has minimal impact on the building load. Therefore, the influence of internal walls and internal floors is ignored during the simulation process. The external wall of the building is comprised of five layers, namely the external coating layer, the ventilated air layer, the insulation layer, the structural layer, and the internal wall surface, from outermost to innermost, with a total thickness of 250 mm. The roof of the building consists of seven layers, arranged from outside to inside as the pebble layer, protective membrane, insulation layer, waterproof layer, cement mortar leveling layer, lightweight aggregate concrete sloping layer, and reinforced concrete roof slab, with a total thickness of 270 mm. Initially, the design incorporated single-glazed glass windows (6 mm thick). However, for comparison and evaluation purposes, double-glazed glass windows (6 + 12 + 6 mm) and triple-glazed glass windows (6 + 12 + 6 + 12 + 6 mm) are being considered as alternative options. The shading coefficient of windows is set as 0.5, indicating that half of the solar radiation energy penetrates the room, considering that most buildings of this kind in China are fitted with curtains or external shading devices. Additionally, when the air conditioning is on and the windows are closed, the fresh air load is calculated based on a fresh air volume of 30 m³/h per person, yielding an air change rate of approximately 0.2 times per hour. Considering the daily routines of the elderly, supplementary lighting is provided from 7:00 AM to 8:00 PM at a power of 7 W/m². Outside these hours, the elderly are presumed to be resting, with no whole-house lighting, and its power consumption is deemed negligible. Given potential mobility issues among the elderly, who spend most of their time indoors, it is assumed that there are always 16 people present in the room. The Group Coefficient is a crucial parameter utilized in Chinese standards for the calculation of human body heat load. The human body heat load is determined using Eq. (2), with $\:{\varvec{C}}_{{\mathbf{c}\mathbf{l}}_{\mathbf{r}\mathbf{t}}}$ set to 1 when personnel are present indoors for 24 h. The group coefficient, which varies depending on the type of building occupied by the personnel, acts as a correction factor as specified in the standard.

2 $$\:{Q}_{\text{P}}={C}_{{\text{c}\text{l}}_{\text{r}\text{t}}}\phi\:{Q}_{\text{r}\text{t}}$$

In which, the $\:{\varvec{C}}_{{\mathbf{c}\mathbf{l}}_{\mathbf{r}\mathbf{t}}}$ represent the human body cooling load coefficient, representing the correction for the duration of personnel presence in the room on the body cooling load of people. The *φ* represent the group coefficient, which varies depending on the type of building occupied by the personnel. The *Q*_rt_ represent the human heat dissipation.

**Table 2:** The paramenters for simulation.

Items	Value
Heat transfer coefficient of the external walls (W·m^− 2^·°C ^− 1^)	0.5
Thicknesses of the external walls (m)	0.25
Heat transfer coefficient of the roof (W·m^− 2^·°C ^− 1^)	0.5
Thicknesses of the roof (m)	0.27
Heat transfer coefficient of the window (W·m^− 2^·°C ^− 1^)	4.0
Thermal conductivity of EPS (W·m^− 1^·°C ^− 1^)	0.038
Convection heat transfer coefficient between wall surface and indoor air (W·m^− 2^·°C ^− 1^)	8.7
Convection heat transfer coefficient between wall surface and outdoor air (W·m^− 2^·°C ^− 1^)	5.7 + 3.8*v*
Heat transfer coefficient of single-glazed glass (W·m^− 2^·°C ^− 1^)	4.0
Heat transfer coefficient of double-glazed glass (W·m^− 2^·°C ^− 1^)	1.3
Heat transfer coefficient of triple-glazed glass (W·m^− 2^·°C ^− 1^)	0.6
Transmittance of single-glazed glass	0.92
Transmittance of double-glazed glass	0.83
Transmittance of triple-glazed glass	0.73
Shading coefficient of windows	0.5
Group coefficient	0.98
Ventilation rate when the air conditioning is on (m^3^/(h·1person))	30
Heat dissipation of equipment (W/m^2^)	13
Heat dissipation of lighting (W/m^2^) (from 7:00am to 8:00pm)	7

### Economic assessment method

This study mainly focuses on the renovation of existing buildings. It is assumed that additional investments are made to improve the thermal insulation performance of walls and windows based on the existing building envelope, thereby reducing the cooling and heating loads of the building, thus reduce the operating costs. The lifespan of insulation materials is 25(± 5) years. During this period, the operating costs of air conditioning for cooling and heating, and the initial investment of insulation materials, together constitute the full life cycle cost of temperature regulation (Eq. 3). The existing external wall of the building has a total heat transfer coefficient of 0.5 W·m^− 2^·°C ^− 1^, which is set as the benchmark (0RMB). Based on this, the additional investment in insulation materials or windows is set as the initial cost (Eq. 4). The operating costs for different enclosure structures are calculated by taking into account the electricity consumption of air conditioning, which is determined by the cooling and heating loads, and the COP of the air conditioner (Eq. 5).

3 $${\text{LCC}}\,=\,{\text{IC}}\,+\,{\text{OC}}$$

4 $$IC=d\,{\cdot}\,S\,{\cdot}\,p\,{\cdot}\,P_i$$

5 $$\:\text{O}\text{C}=\frac{\sum\:(\left|{Q}_{0}\right|{\cdot}t)}{\text{C}\text{O}\text{P}}{\cdot}{P}_{\text{e}}$$

In which, the LCC represent the life cycle cost, RMB; the IC represent the initial cost, RMB; the OC represent the operating cost, RMB; *d* represent the thickness of the wall insulation material, m; S represent the area of the wall insulation material, m^2^; *ρ* represent the density of the wall insulation material, kg/m^3^; *P*_i_ represent the unit cost of the insulation material, RMB/kg; *t* represent the time, h; COP represent the coefficient of performance of the air conditioning, which is assumed as 3; *P*_e_ represent the unit cost of the electric power, RMB/(kW·h).

It is important to acknowledge the limitations inherent in any cost analysis model, particularly when dealing with long-term projections over a 25-year horizon. Energy price fluctuations, influenced by complex and often unpredictable factors such as geopolitical tensions and inflation rates, present a significant challenge to the accuracy of such models. In this study, the analysis is intentionally simplified by assuming static energy costs, primarily to focus on the relative performance and cost-effectiveness of different insulation materials and glazing systems under current market conditions. This approach allowed us to isolate the impact of these building components on energy consumption and costs, without being overshadowed by external economic factors. However, energy prices are likely to vary, and this variability could impact the overall life cycle costs. Similarly, while our assumptions about maintenance costs are based on the average rated lifespan of the materials and the typical maintenance requirements observed in practice, it is possible that unforeseen circumstances could necessitate earlier replacements or additional maintenance. These limitations are inherent in any long-term cost analysis and should be taken into account when interpreting the results.

Through market research in China, it has been found that the price of EPS is 11 RMB/kg. The cost of double-glazed glass is 100 RMB/m^2^ higher than that of single-glazed glass, and the cost of triple-glazed glass is an additional 100 RMB/m^2^ higher than that of double-glazed glass windows. The unit cost of the electric power is 0.5 RMB/(kW·h). The prices for insulation materials and windows are obtained through comprehensive market research, involving direct inquiries with multiple suppliers both online and offline, as well as telephone consultations. To avoid potential biases and maintain research integrity, specific supplier information is not disclosed. Instead, averaged and normalized prices that reflect current market conditions are presented. Similarly, the electricity prices used in the analysis are sourced from official local utility rates, assuming a stable rate over the projection period for simplicity.

## Results

### Heat and cold load characteristics of the five cities in hot summer and cold winter area

**Fig. 4:**
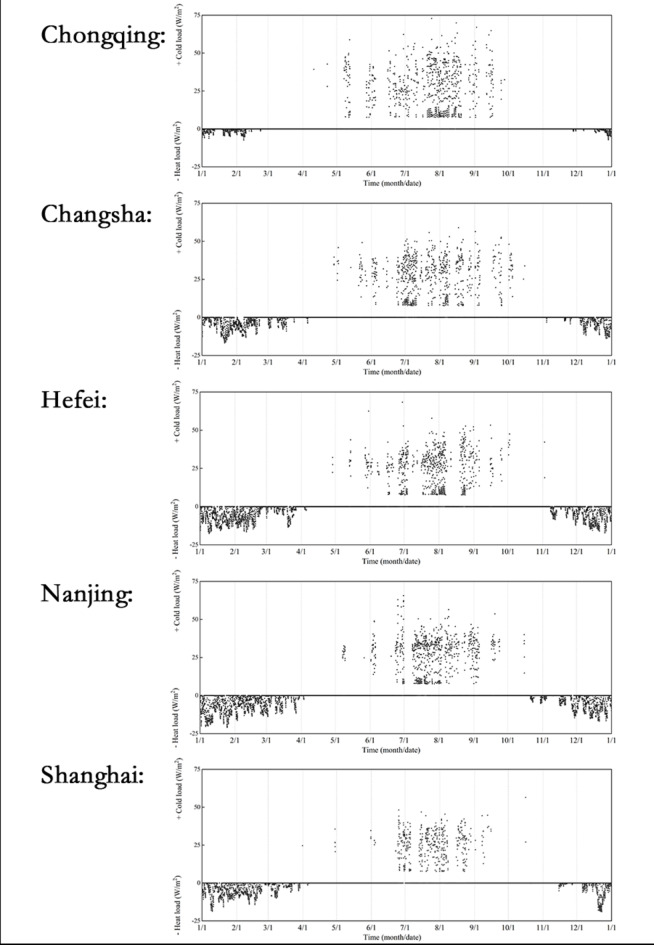
The houly heat load and cold load of the five cities.

Under typical operating conditions, the hourly cooling and heating loads for five towns over a period of 8760 h (year-round) are shown in Fig. 4. The unit of the vertical axis is W/m², representing the cooling or heating load per square meter of building area.

As can be seen from the Fig. 4, the cooling load in the hot summer and cold winter area is mainly concentrated in the months of June, July, August, and September. There is a certain gap between the minimum value of the cooling load and the horizontal axis, which is due to the energy-saving strategy of using natural ventilation by opening windows in senior living community buildings. When the outdoor temperature is lower than the indoor set temperature, the cooling load is eliminated through window ventilation. However, when the outdoor temperature is higher than the indoor set temperature, the heat transfer from the building envelope structure combined with internal sources of heat inside the room results in a building load much higher than zero. Besides, the simulated summer heating loads in this study are significantly higher than those in winter, which can be attributed to the meteorological parameters of the hot summer and cold winter region. Furthermore, the continuous heat emission from occupants and equipment, coupled with the solar radiation heating the rooms during the day, results in the heating load being greater than the cooling load.

The heating load is mainly concentrated in January, February, March, and December. Among them, Hefei and Nanjing have a longer heating load duration, whereas Chongqing has a shorter one. The maximum summer cooling load for Chongqing, Changsha, Hefei, Nanjing, and Shanghai is 72.9 W/m^2^, 58.9 W/m^2^, 66.8 W/m^2^, 65.7 W/m^2^, and 56.5 W/m^2^ respectively, while the maximum winter heating load is -7.4 W/m^2^, -16.9 W/m^2^, -17.6 W/m^2^, -20.9 W/m^2^, and − 18.9 W/m^2^, respectively. In the hot summer and cold winter area, senior living community buildings have a much higher summer cooling load than winter heating load, which is due to the lower optimal comfortable temperature for senior people in winter. As senior people tend to wear thick winter clothes for warmth, the temperature set for air conditioning during heating is lower, resulting in a smaller temperature difference between inside and outside the room. In addition, the differences in local meteorological parameters between winter and summer, solar radiation, and human thermal load all contribute to the heating load being greater than the cooling load.

**Fig. 5:**
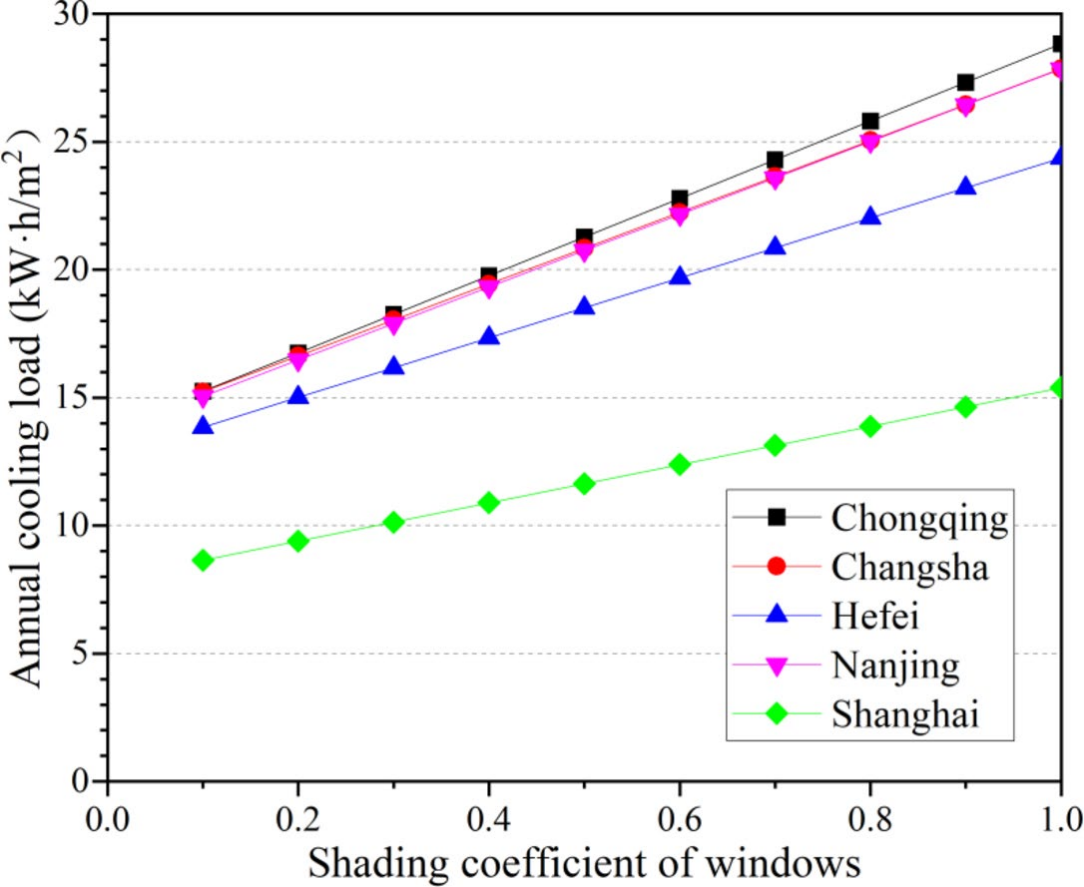
The effect of window shading coefficients on annual cooling loads.

In summer, external building shading significantly impacts cooling loads, and most households adopt shading strategies to reduce air conditioning energy consumption. The shading coefficient is used to quantify the extent of external shading, where a smaller shading coefficient indicates better performance in blocking solar heat gain from entering indoors. When no external shading is applied, the shading coefficient is set to 1. Therefore, Fig. 5 illustrates the effect of window shading coefficients on annual cooling loads.

As shown in Fig. 5, the annual cooling load gradually increases with an increase in the shading coefficient (indicating stronger shading effectiveness), but the increase magnitudes vary across cities. When the shading ratio decreases from 1 (no shading) to 0.1 (maximum shading), the air conditioning loads in Chongqing, Changsha, Hefei, Nanjing, and Shanghai decrease by 47.1%, 45.3%, 43.2%, 46.0%, and 43.8%, respectively, highlighting the critical role of external shading. However, a shading ratio of 0.5 is pragmatically chosen as the optimal value for most households. Excessively low shading ratios (e.g., below 0.5) may lead to insufficient daylighting and psychological discomfort due to overly dim interiors. Thus, all subsequent analyses in this study are based on a shading ratio of 0.5.

**Fig. 6:**
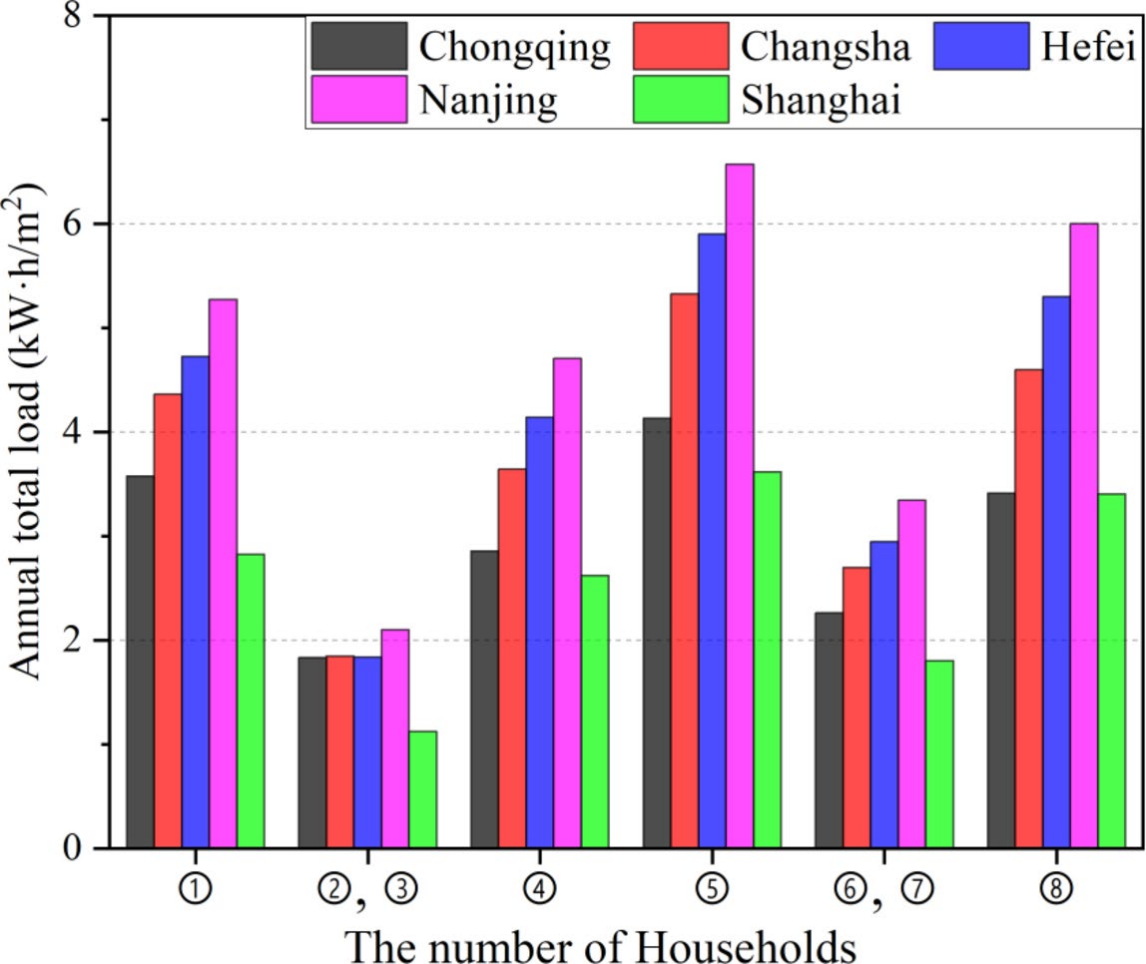
The annual total load differences among households in different locations.

Figure 6 illustrates the annual total load differences among households in different locations. It can be observed that households with larger exterior facade areas exhibit higher annual total loads. households ⑤ and ⑧, located on the western and eastern sides of the second floor, respectively, have the highest annual loads because they have the largest contact area with the external environment, including east/west exterior walls and the roof. Next are the first-floor households on the eastern and western sides(Households ① and ④), whose exterior wall areas are larger than those of the remaining four households. Among the four households without exterior walls, those with roofs (Households 6 and 7) have higher annual loads than those without roofs (Household 2 and 3). In the same city, the load of the highest-consumption household can be more than twice as large as that of the lowest-consumption household (e.g., Hefei, Nanjing, and Shanghai). Comparing differences across cities, the annual loads rank from highest to lowest as Nanjing, Hefei, Changsha, Chongqing, and Shanghai, which correlates with their unique climatic characteristics.

**Fig. 7:**
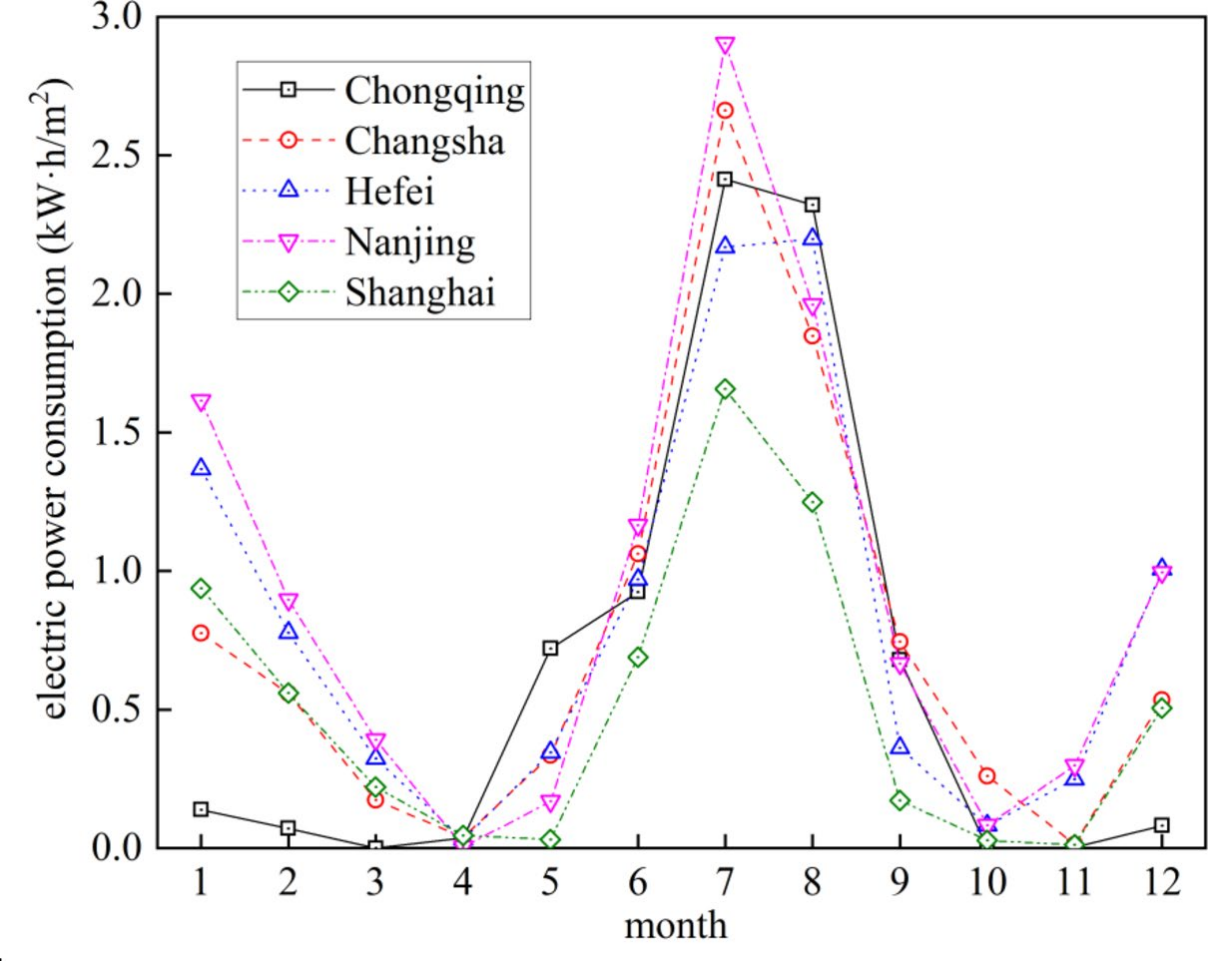
Monthly electirc power cosumption of the five cities.

Figure 7 shows the monthly electric power consumption of the five cities. The unit of the vertical axis represents the monthly total load per square meter of building area. From the figure, it can be seen that the electric power consumption of the building is relatively low in March, April, and October-November, due to the comfortable outdoor temperature. The electric power consumption in the hotest month (July) of the five cities ranges from 1.6 kW·h/m^2^ to 2.9 kW·h/m^2^, ranked from highest to lowest as: Nanjing, Changsha, Chongqing, Hefei, and Shanghai. In winter, the electric power consumption in the coldest month (January) of the five cities ranges from 0.1 kW·h/m^2^ to 1.7 kW·h/m^2^, ranked from highest to lowest as Nanjing, Hefei, Shanghai, Changsha, and Chongqing. Chongqing is located in the inland area with many mountains surrounding it, so cold currents pass through it less frequently during winter, resulting in a very small amount of cooling load.

### The impact of increasing the thickness of wall insulation

The additional thickness represents the extra insulation that has been applied over the existing wall structure. The original wall has a thermal conductivity of 0.5 W·m^− 2^·°C^− 1^. The increased insulation material is located in the insulation layers of both the walls and the roof. The thickness shown in the figure represents an increment based on the retrofitting of an existing building. The recommended insulation thickness values are determined by comparing the LCC of different insulation thicknesses. For each city, the insulation thickness corresponding to the lowest cost is identified as the optimal choice. Noting that the typical insulation thickness for ordinary buildings in China generally falls between 0.05 m and 0.1 m, this study expands the range to 0 m to 0.2 m and selects 11 insulation thicknesses at 0.02 m intervals for a comprehensive comparison. To ascertain the cost associated with each thickness, the insulation thickness is inputted into a building load calculation model to obtain hourly loads and electricity consumption throughout the year, followed by summing the annual electricity consumption to calculate the air conditioning operating cost, OC, and LCC.

**Fig. 8:**
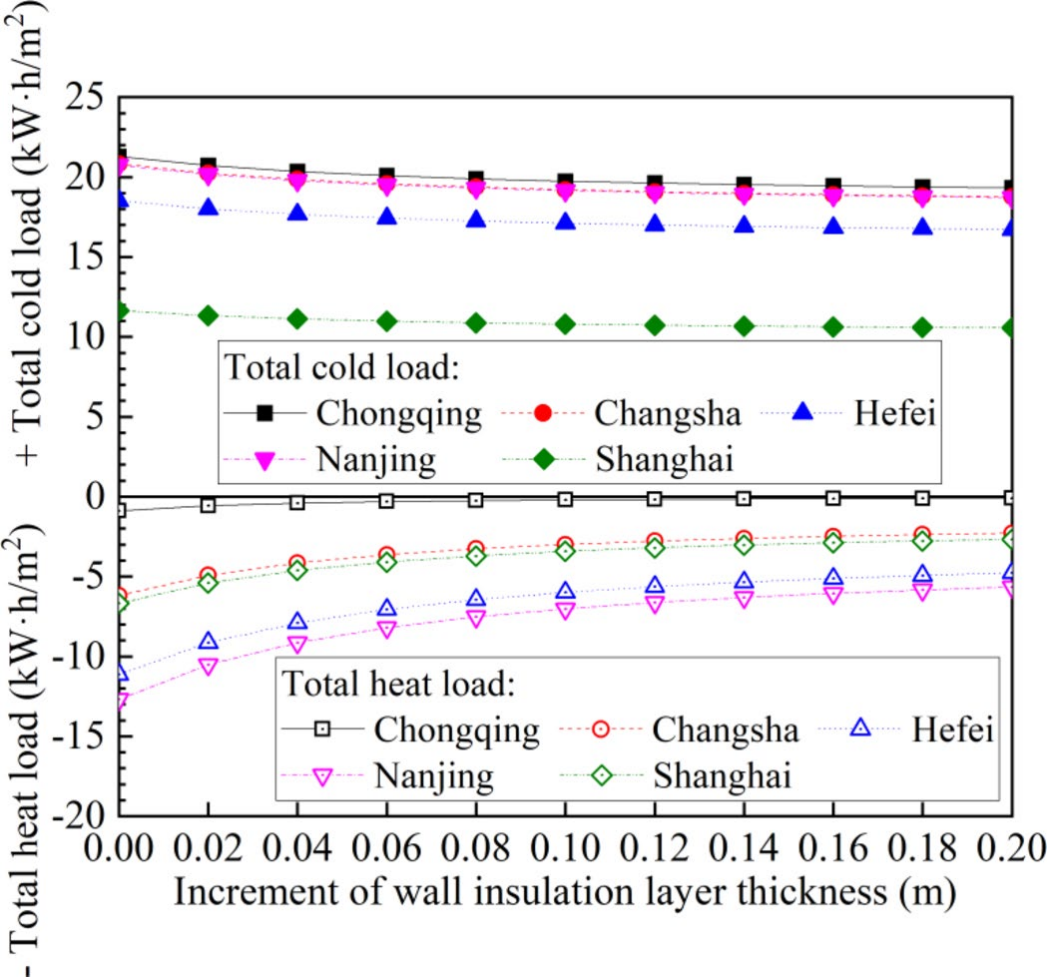
Annual heat and cold load vary with the increment of wall insulation layer thickness for the five cities.

As the increment of wall insulation layer thickness increases, the cold and heat loads in five cities continue to decrease, as shown in Fig. 8. When the insulation layer thickness is initially increasing, the heat and cold load change rate is relatively fast; however, when the insulation layer thickness is large, the rate of load change is relatively small. When the insulation layer thickness increases to 0.2 m, the cold load of the five cities decreases by 9.2–9.9%, while the heat load decreases by 5.7–88.7%.

With the increase of wall insulation layer thickness, the initial investment shows a linear increase. The corresponding relationship between them is shown in Table 3.

**Table 3:** The initial investment varies with the increment of wall insulation layer thickness.

The increment of wall insulation layer thickness	The initial investment	The increment of wall insulation layer thickness	The initial investment	The increment of wall insulation layer thickness	The initial investment
0.00 m	0 RMB	0.08 m	8601 RMB	0.16 m	17,202 RMB
0.02 m	2150 RMB	0.10 m	10,751 RMB	0.18 m	19,353 RMB
0.04 m	4301 RMB	0.12 m	12,902 RMB	0.20 m	21,503 RMB
0.06 m	6451 RMB	0.14 m	15,052 RMB		

**Fig. 9:**
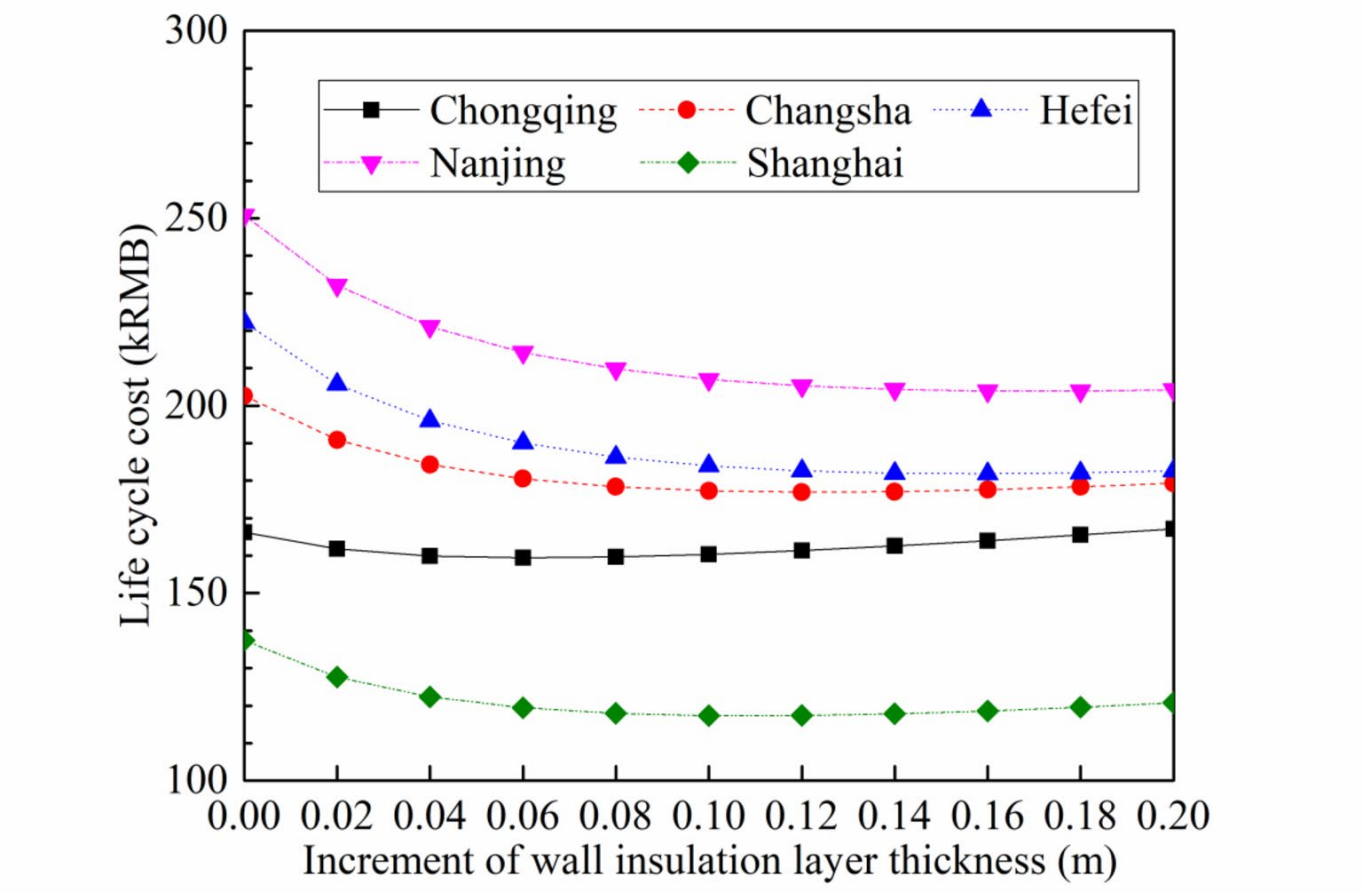
Life cycle cost vary with the increment of wall insulation layer thickness for the five cities.

The relationship between the increment of wall insulation layer thickness and life cycle cost in the five cities is shown in Fig. 9. As the insulation layer thickness increases, the cost first decreases and then increases. However, the optimal insulation layer thickness for each of these five towns is different, and they are: Chongqing − 0.06 m, Changsha − 0.12 m, Hefei − 0.16 m, Nanjing − 0.16 m, Shanghai − 0.10 m. The optimal insulation layer thickness for Chongqing, which is located on the westernmost edge of the warm-cold region, is the smallest because the winter temperatures in Chongqing are low and the demand for heating is low, resulting in the minimum annual electric power consumption. In contrast, Hefei and Nanjing have the higher annual electric power consumption and require thicker insulation layer thicknesses to reduce operating costs.

### The impact of glass types

**Fig. 10:**
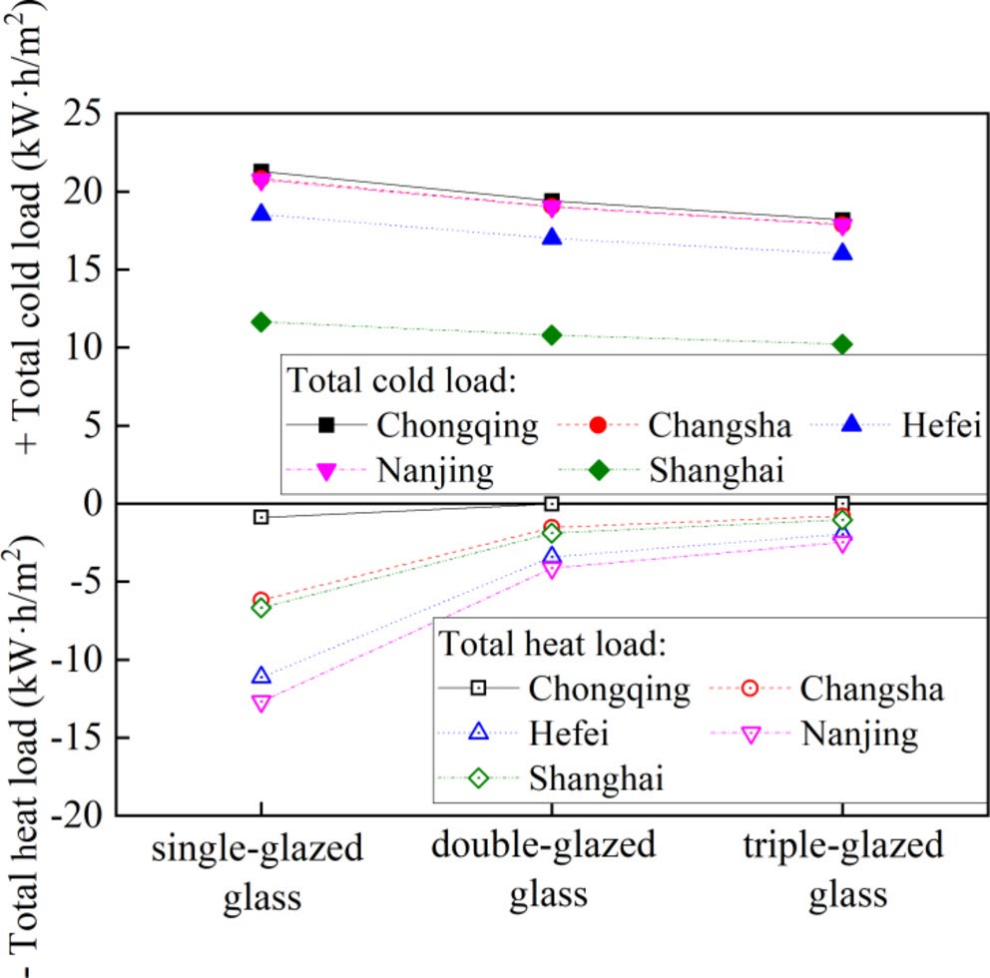
Annual heat load and cold load vary with the glass type for the five cities.

As the number of the glass increases, the cold and heat loads in five cities continue to decrease, as shown in Fig. 10. When the glass changes from single-glazed to tripl-glazed, the cold load of the five cities decreases by 12.3–14.6%, while the heat load decreases by 82.4–99.6%. This shows that the way of improving the glass has a greater impact on the building heat and cold load than that of improving the thickness of the wall insulation layer thickness.

**Fig. 11:**
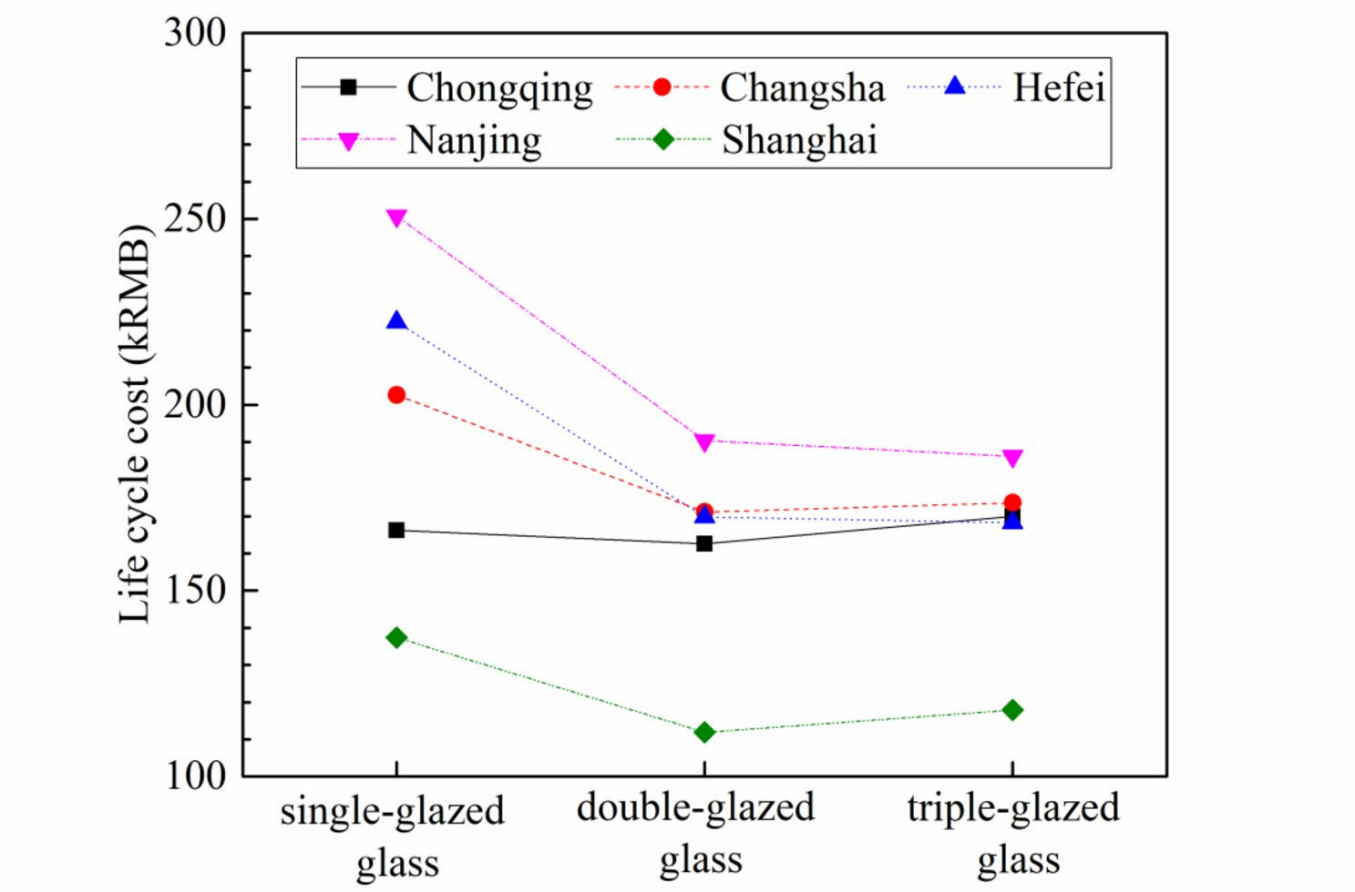
Life cycle cost vary with the glass type for the five cities.

The relationship between the glass type and life cycle cost in the five cities is shown in Fig. 11, when the heat transfer coefficient of the walls is 0.5 W·m^− 2^·°C ^− 1^. When using double-glazed glass or triple-glazed glass, the initial investment increases by 16.8kRMB or 33.6kRMB compared to single-glazed glass, respectively. The optimal type of window varies from town to town, with double-glazed glass suitable for Chongqing, Changsha, and Shanghai, while triple-glazed glass are preferred in Hefei and Nanjing due to their higher annual total load compared to the other towns.

### Comprehensive optimization of wall insulation layer thickness and glass type

The thickness of the wall insulation layer and the type of glass both have a significant impact on the air conditioning energy consumption of a building, and how to choose between them in practical engineering is crucial. Therefore, in this section, the impact of different combinations of wall thickness and window type on annual heat and cold loads and life cycle cost is simulated, in search of the optimal parameters for different cities.

**Fig. 12:**
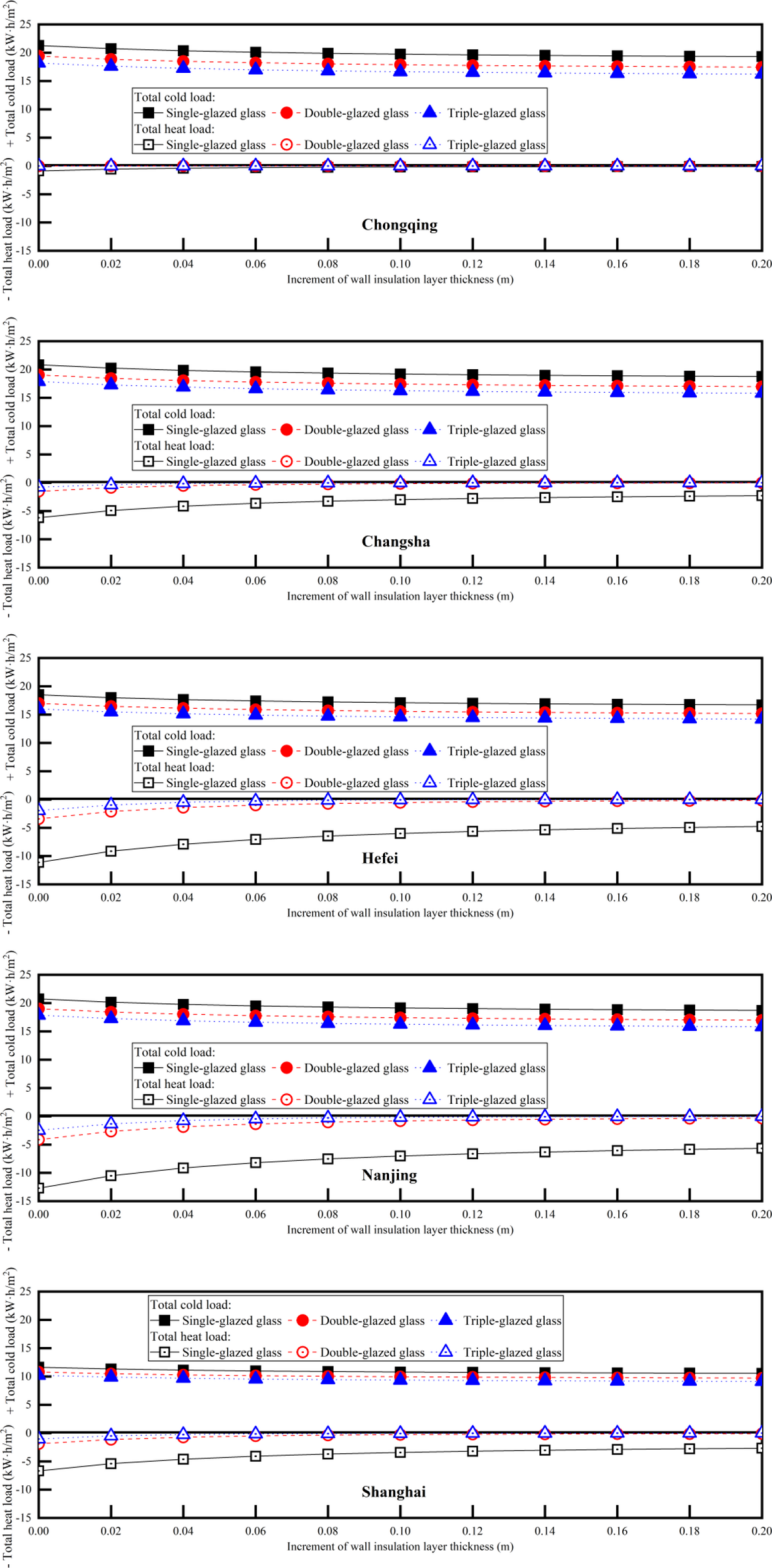
Annual heat load and cold load vary with the increment of wall thickness and glass type.

In the search for optimal parameters tailored to different cities, an analysis of cold and heat loads with varying wall insulation layer thicknesses and glass type, as depicted in Fig. 12. As the insulation layer thickens or the number of glass layers increases, both cold and heat loads experience a decline. Notably, Chongqing exhibits the highest cooling load, contrasting with Shanghai’s lowest. Conversely, Nanjing tops the chart for thermal loads, while Chongqing has the smallest. Furthermore, upgrading windows from single-glazed glass to double-glazed glass results in a marked reduction in both cooling and heating loads, with the heating load decrease being particularly noteworthy. The improvement, however, becomes less pronounced when switching from double-glazed to triple-glazed glass. The total cold load and total heat load are relatively balanced for Changsha, Hefei, Nanjing, and Shanghai, despite the peak cooling load in summer exceeding the minimum heating load in winter. This phenomenon is influenced by the data distribution, as shown in Fig. 3. Specifically, the data points for summer cooling loads are sparser, while those for winter heating loads are denser. This balance can be explained by behavioral patterns related to window usage. During cooler summer nights, opening windows increases air convection, which reduces cooling loads. In contrast, during winter, reducing heating loads through ventilation is not as feasible.

**Fig. 13:**
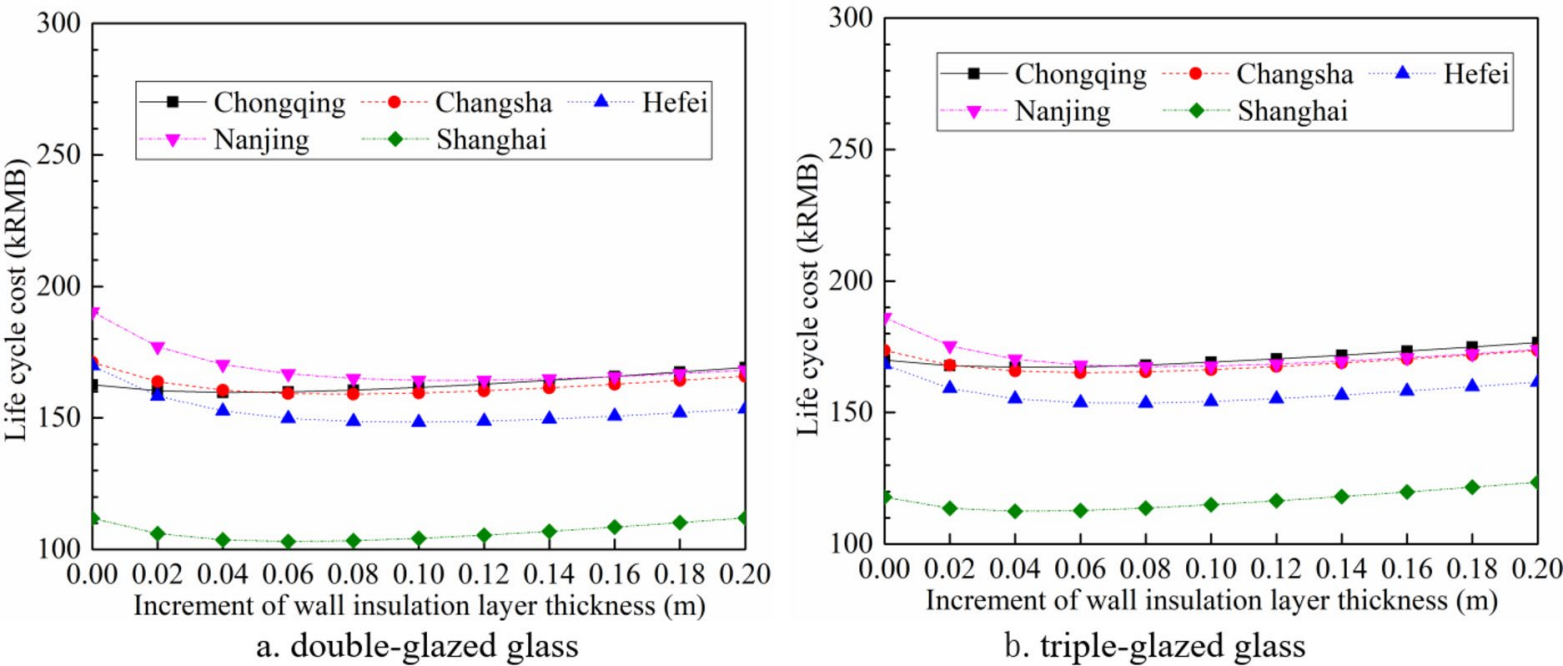
Life cycle cost vary with the increment of wall thickness for double-glazed glass and triple-glazed glass.

When the glass type is double-glazed or triple-glazed, the LCC variation with the wall insulation layer thickness can be seen in Fig. 13. As comparied with Fig. 7, the lowest LCC for all cities except Chongqing correspond to double-glazing. The corresponding insulation layer thicknesses are: Changsha-0.08 m, Hefei-0.10 m, Nanjing-0.10 m, and Shanghai-0.06 m. Compared to double-glazing, the minimum LCC of single-glazed glass and triple-glazed glass are 11.2-24.1% and 1.9-9.2% higher, respectively. For Chongqing, the lowest LCC corresponds to single-glazing with an insulation layer thickness of 0.06 m, which is 0.2% and 4.9% less than the minimum LCC of double-glazed glass and triple-glazed glass, respectively.

## Conclusion

This paper focuses on the characteristics of senior living community buildings and selects five representative towns in the hot summer and cold winter area to simulate and calculate the impact of wall insulation layer thickness and glass type on building cooling and heating load and life cycle cost (LCC). The conclusions are as follows:

Firstly, with the increase in insulation layer thickness or the number of window layers, building loads continue to decrease. Due to the fact that winter heat loads are much smaller than summer heat loads in the hot summer and cold winter area, the proportion of heat load reduction with increased envelope strength is greater than that of cooling load changes.

Secondly, under certain conditions of glass type, with increasing wall insulation layer thickness, the LCC of senior living community buildings shows a trend of decreasing first and then increasing, indicating the existence of an optimal insulation layer thickness.

Thirdly, for Chongqing, the optimal glass type for minimizing LCC is single-glazed, with a wall insulation layer thickness of 0.06 m. For Changsha, Hefei, Nanjing, and Shanghai, the optimal glass type for minimizing LCC is double-glazed, with wall insulation layer thicknesses of 0.08 m, 0.10 m, 0.10 m, and 0.06 m, respectively.

Forthly, comparing these five cities, Shanghai has the lowest LCC, which is approximately 30% lower than the other four cities due to its relatively smaller cooling load in the summer. The minimum LCC for the remaining four cities is between 148kRMB and 165kRMB.

This study primarily focuses on the renovation of buildings in senior living communities situated in the hot summer and cold winter regions of China., specifically discussing optimization schemes for walls and windows. Several limitations should be noted:

Firstly, the simulation employs a steady-state heat conduction model that neglects thermal mass effects of building materials. These assumptions may inevitably introduce certain deviations into the simulation results.

Secondly, the load calculations treat the building’s internal environment as a unified whole without considering functional zoning. Implementing differentiated environmental control strategies for specific areas (e.g., balconies and storage rooms) or developing partition-specific wall/window configurations and shading strategies could potentially reduce building loads and alter optimal retrofitting conclusions.

Thirdly, The calculations conducted are grounded in the assumptions of an external wall thermal conductivity of 0.5 W·m-2·°C-1 and a window-to-wall ratio set at 0.236. Notably, variations in initial wall parameters may require further parameter adjustments or conversion methods.

Despite these limitations, the qualitative conclusions presented maintain broad applicability and aim to inform practical engineering projects. Future work will seek to identify more universal principles to guide retrofitting practices for elderly-oriented communities.

## Data Availability

The datasets generated and/or analysed during the current study are available in the [ENERGYPLUS] repository, [https://energyplus.net/weather-region/asia_wmo_region_2/CHN].

## List of symbols

Q_0_: Heat load or cool load of the building
*Q*_glass_: Heat transfer by radiation through the glass, W
*Q*_air_: Heat dissipation by ventilation and infiltration, W
*Q*_S_: Heat dissipation by lighting, W
$$\varphi$$: Group coefficient
LCC: Life cycle cost, RMB
OC: Operating cost, RMB
S: Area of the wall insulation material, m^2^
*P*_i_: Unit cost of the insulation material, RMB/kg
COP: Coefficient of performance of the air conditioning
*Q*_body_: Heat transfer by the envelope, W
*Q*_P_: Body cooling load of people, W
*Q*_E_: Heat dissipation by equipment, W
$\:{\varvec{C}}_{{\mathbf{c}\mathbf{l}}_{\mathbf{r}\mathbf{t}}}$: Human body cooling load coefficient
*v*: Wind speed, m/s
IC: Initial cost, RMB
*d*: Thickness of the wall insulation material, m
*p*: Density of the wall insulation material, kg/m^3^
*t*: Time, h
*P*_e_: The unit cost of the electric power, RMB/(kW·h).
